# 1-[6-Chloro-4-(2-chloro­phen­yl)-2-methyl-3-quinol­yl]ethanone

**DOI:** 10.1107/S1600536810026991

**Published:** 2010-07-14

**Authors:** B. Preeti, S. Sarveswari, V. Vijayakumar, Kang Wai Tan, Edward R. T. Tiekink

**Affiliations:** a Organic Chemistry Division, School of Advanced Sciences, VIT University, Vellore 632 014, India; b Department of Chemistry, University of Malaya, 50603 Kuala Lumpur, Malaysia

## Abstract

The title compound, C_18_H_13_Cl_2_NO, features an essentially planar quinoline ring system (r.m.s. deviation = 0.023 Å) with the acetyl [C—C—C—O torsion angle = −78.27 (17)°] and benzene [C—C—C—C torsion angle = 110.11 (14)°] substituents being twisted out of the plane; the dihedral angle formed between the mean planes of these two substituents is 58.01 (8)°. The acetyl O and benzene-bound Cl atoms lie to opposite sides of the mol­ecule. Centrosymmetric aggregates mediated by pairs of C—H⋯O contacts are found in the crystal structure, and these are connected into a two-dimensional array in the (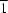
01) plane *via* Cl⋯O [3.0508 (11) Å] inter­actions.

## Related literature

For background to the pharmaceutical potential of quinoline derivatives, see: Musiol *et al.* (2006 ▶). For related structures, see: Kaiser *et al.* (2009 ▶); Viji *et al.* (2010 ▶). For a review on halogen bonding, including short halogen⋯oxygen inter­actions, see: Fourmigué (2009 ▶). 
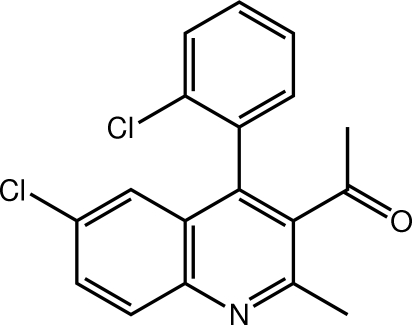


## Experimental

### 

#### Crystal data

C_18_H_13_Cl_2_NO
*M* *_r_* = 330.22
Monoclinic, 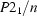

*a* = 10.3105 (6) Å
*b* = 12.8882 (7) Å
*c* = 11.7968 (7) Å
β = 93.367 (1)°
*V* = 1564.90 (16) Å^3^
*Z* = 4
Mo *K*α radiation
μ = 0.42 mm^−1^
*T* = 100 K
0.29 × 0.24 × 0.19 mm

#### Data collection

Bruker SMART APEX diffractometer
Absorption correction: multi-scan (*SADABS*; Sheldrick, 1996 ▶) *T*_min_ = 0.933, *T*_max_ = 1.000
14786 measured reflections
3594 independent reflections
3189 reflections with *I* > 2σ(*I*)
*R*_int_ = 0.025

#### Refinement

*R*[*F* ^2^ > 2σ(*F* ^2^)] = 0.029
*wR*(*F* ^2^) = 0.079
*S* = 1.03
3594 reflections
201 parameters
H-atom parameters constrained
Δρ_max_ = 0.38 e Å^−3^
Δρ_min_ = −0.24 e Å^−3^

### 

Data collection: *APEX2* (Bruker, 2008 ▶); cell refinement: *SAINT* (Bruker, 2008 ▶); data reduction: *SAINT*; program(s) used to solve structure: *SHELXS97* (Sheldrick, 2008 ▶); program(s) used to refine structure: *SHELXL97* (Sheldrick, 2008 ▶); molecular graphics: *ORTEP-3* (Farrugia, 1997 ▶) and *DIAMOND* (Brandenburg, 2006 ▶); software used to prepare material for publication: *publCIF* (Westrip, 2010 ▶).

## Supplementary Material

Crystal structure: contains datablocks global, I. DOI: 10.1107/S1600536810026991/lh5080sup1.cif

Structure factors: contains datablocks I. DOI: 10.1107/S1600536810026991/lh5080Isup2.hkl

Additional supplementary materials:  crystallographic information; 3D view; checkCIF report

## Figures and Tables

**Table 1:** Hydrogen-bond geometry (Å, °)

*D*—H⋯*A*	*D*—H	H⋯*A*	*D*⋯*A*	*D*—H⋯*A*
C18—H18⋯O1^i^	0.95	2.59	3.2460 (17)	127

Symmetry code: (i) 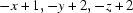.

**Fig. 1.**
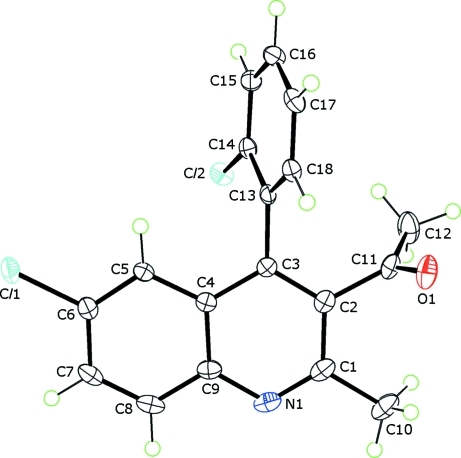
The molecular structure of (I) showing displacement ellipsoids at the 50% probability level.

**Fig. 2.**
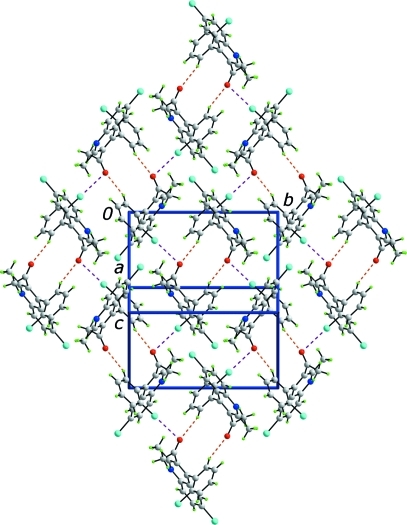
2-D array formed in the (1 0 1) plane in (I) mediated by C–H···O and Cl···O contacts shown as orange and purple dashed lines, respectively.

## supplementary crystallographic information

### Comment

On-going structural studies of quinoline derivatives (Kaiser *et al.*, 2009; Viji *et al.*, 2010) are motivated by their potential pharmacological properties (Musiol *et al.*, 2006). Herein, the crystal and molecular structure of the title compound (I) is described.

The non-hydrogen atoms comprising the quinoline nucleus in (I) are planar [r.m.s. deviation = 0.023 Å]. Each of the attached acetyl and benzene groups are twisted out of the plane of the quinoline residue as seen in the values of the C1–C2–C11–O1 and C2–C3–C13–C14 torsion angles of -78.27 (17) and 110.11 (14) °, respectively. The acetyl group and benzene ring are non-parallel with the dihedral angle between their least-squares planes being 58.01 (8) °. The acetyl-O and benzene-Cl atoms lie to opposite sides of the molecule.

The most prominent intermolecular interactions in the crystal structure are of the type C–H···O, Table 1, and Cl···O (Fourmigué, 2009). The C–H···O contacts lead to the formation of centrosymmetric dimers and these are connected into a 2-D array in the (1 0 1) plane by Cl···O interactions [Cl2···O1^i^ = 3.0508 (11) Å for -1/2 + *x*, 3/2 - *y*, -1/2 + *z*].

### Experimental

A mixture of 2-amino-2',5-dichlorobenzophenone (0.01 *M*), acetylacetone (0.01 *M*) and a catalytic amount of conc. HCl was irradiated under 240 W for about 6 min. The resultant solid was filtered, dried and purified by column chromatography using a 1:1 mixture of ethyl acetate and petroleum ether and recrystallized using ethanol. *M*. pt. 389 K. Yield: 58%

### Refinement

Carbon-bound H-atoms were placed in calculated positions (C—H 0.95 to 0.98 Å) and were included in the refinement in the riding model approximation, with *U*_iso_(H) set to 1.2 to 1.5*U*_equiv_(C).

### Crystal data

**Table:**

C_18_H_13_Cl_2_NO	*F*(000) = 680
*M**_r_* = 330.22	*D*_x_ = 1.402 Mg m^−^^3^
Monoclinic, *P*2_1_/*n*	Mo *K*α radiation, λ = 0.71073 Å
Hall symbol: -P 2yn	Cell parameters from 6804 reflections
*a* = 10.3105 (6) Å	θ = 2.3–28.2°
*b* = 12.8882 (7) Å	µ = 0.42 mm^−^^1^
*c* = 11.7968 (7) Å	*T* = 100 K
β = 93.367 (1)°	Block, colourless
*V* = 1564.90 (16) Å^3^	0.29 × 0.24 × 0.19 mm
*Z* = 4	

### Data collection

**Table:**

Bruker SMART APEX diffractometer	3594 independent reflections
Radiation source: fine-focus sealed tube	3189 reflections with *I* > 2σ(*I*)
graphite	*R*_int_ = 0.025
ω scans	θ_max_ = 27.5°, θ_min_ = 2.3°
Absorption correction: multi-scan (*SADABS*; Sheldrick, 1996)	*h* = −13→13
*T*_min_ = 0.933, *T*_max_ = 1.000	*k* = −16→16
14786 measured reflections	*l* = −15→15

### Refinement

**Table:**

Refinement on *F*^2^	Primary atom site location: structure-invariant direct methods
Least-squares matrix: full	Secondary atom site location: difference Fourier map
*R*[*F*^2^ > 2σ(*F*^2^)] = 0.029	Hydrogen site location: inferred from neighbouring sites
*wR*(*F*^2^) = 0.079	H-atom parameters constrained
*S* = 1.03	*w* = 1/[σ^2^(*F*_o_^2^) + (0.0409*P*)^2^ + 0.6548*P*] where *P* = (*F*_o_^2^ + 2*F*_c_^2^)/3
3594 reflections	(Δ/σ)_max_ = 0.001
201 parameters	Δρ_max_ = 0.38 e Å^−^^3^
0 restraints	Δρ_min_ = −0.24 e Å^−^^3^

### Special details

**Table:**

Geometry. All s.u.'s (except the s.u. in the dihedral angle between two l.s. planes) are estimated using the full covariance matrix. The cell s.u.'s are taken into account individually in the estimation of s.u.'s in distances, angles and torsion angles; correlations between s.u.'s in cell parameters are only used when they are defined by crystal symmetry. An approximate (isotropic) treatment of cell s.u.'s is used for estimating s.u.'s involving l.s. planes.
Refinement. Refinement of *F*^2^ against ALL reflections. The weighted *R*-factor *wR* and goodness of fit *S* are based on *F*^2^, conventional *R*-factors *R* are based on *F*, with *F* set to zero for negative *F*^2^. The threshold expression of *F*^2^ > 2σ(*F*^2^) is used only for calculating *R*-factors(gt) *etc*. and is not relevant to the choice of reflections for refinement. *R*-factors based on *F*^2^ are statistically about twice as large as those based on *F*, and *R*- factors based on ALL data will be even larger.

### Fractional atomic coordinates and isotropic or equivalent isotropic displacement parameters (Å^2^)

**Table:**

	*x*	*y*	*z*	*U*_iso_*/*U*_eq_	
Cl1	−0.23962 (3)	1.07600 (3)	0.72582 (3)	0.02312 (9)	
Cl2	0.13655 (3)	0.82921 (3)	0.61673 (3)	0.02099 (9)	
O1	0.46437 (10)	0.84047 (8)	1.00816 (9)	0.0300 (2)	
N1	0.04450 (12)	0.78055 (9)	1.02995 (9)	0.0227 (2)	
C1	0.17096 (14)	0.76859 (10)	1.02455 (11)	0.0212 (3)	
C2	0.24419 (13)	0.82174 (10)	0.94326 (10)	0.0175 (3)	
C3	0.18344 (12)	0.88978 (9)	0.86805 (10)	0.0150 (2)	
C4	0.04755 (12)	0.90673 (9)	0.87489 (10)	0.0155 (2)	
C5	−0.02314 (12)	0.97804 (10)	0.80428 (10)	0.0160 (2)	
H5	0.0199	1.0186	0.7507	0.019*	
C6	−0.15383 (12)	0.98816 (10)	0.81377 (10)	0.0179 (3)	
C7	−0.22160 (13)	0.92969 (11)	0.89191 (11)	0.0224 (3)	
H7	−0.3128	0.9374	0.8959	0.027*	
C8	−0.15385 (14)	0.86143 (11)	0.96212 (11)	0.0234 (3)	
H8	−0.1987	0.8220	1.0155	0.028*	
C9	−0.01808 (13)	0.84880 (10)	0.95623 (10)	0.0187 (3)	
C10	0.23720 (17)	0.69329 (11)	1.10655 (12)	0.0294 (3)	
H10A	0.1717	0.6565	1.1481	0.044*	
H10B	0.2957	0.7312	1.1604	0.044*	
H10C	0.2874	0.6432	1.0646	0.044*	
C11	0.38793 (14)	0.80115 (10)	0.93929 (11)	0.0209 (3)	
C12	0.42837 (16)	0.72882 (13)	0.84825 (13)	0.0326 (4)	
H12A	0.5219	0.7157	0.8584	0.049*	
H12B	0.4085	0.7603	0.7736	0.049*	
H12C	0.3811	0.6632	0.8532	0.049*	
C13	0.25864 (12)	0.94625 (10)	0.78298 (10)	0.0148 (2)	
C14	0.24475 (12)	0.92459 (10)	0.66698 (10)	0.0159 (2)	
C15	0.31894 (13)	0.97515 (10)	0.58914 (11)	0.0197 (3)	
H15	0.3091	0.9584	0.5107	0.024*	
C16	0.40740 (13)	1.05034 (11)	0.62726 (11)	0.0208 (3)	
H16	0.4582	1.0855	0.5747	0.025*	
C17	0.42194 (13)	1.07433 (10)	0.74183 (12)	0.0200 (3)	
H17	0.4818	1.1265	0.7675	0.024*	
C18	0.34908 (12)	1.02213 (10)	0.81890 (11)	0.0173 (3)	
H18	0.3608	1.0382	0.8974	0.021*	

### Atomic displacement parameters (Å^2^)

**Table:**

	*U*^11^	*U*^22^	*U*^33^	*U*^12^	*U*^13^	*U*^23^
Cl1	0.01579 (16)	0.03008 (18)	0.02304 (17)	0.00616 (12)	−0.00267 (12)	−0.00421 (13)
Cl2	0.02021 (17)	0.02349 (17)	0.01915 (15)	−0.00465 (12)	0.00013 (12)	−0.00655 (12)
O1	0.0281 (6)	0.0284 (5)	0.0319 (5)	0.0051 (4)	−0.0132 (4)	−0.0042 (4)
N1	0.0347 (7)	0.0172 (5)	0.0166 (5)	−0.0016 (5)	0.0055 (5)	0.0005 (4)
C1	0.0338 (8)	0.0147 (6)	0.0150 (6)	0.0018 (5)	−0.0001 (5)	−0.0009 (5)
C2	0.0224 (7)	0.0147 (6)	0.0152 (6)	0.0014 (5)	−0.0015 (5)	−0.0043 (5)
C3	0.0174 (6)	0.0141 (5)	0.0133 (5)	−0.0003 (5)	0.0004 (4)	−0.0027 (4)
C4	0.0173 (6)	0.0146 (6)	0.0148 (5)	−0.0012 (5)	0.0015 (5)	−0.0031 (4)
C5	0.0158 (6)	0.0169 (6)	0.0157 (6)	−0.0009 (5)	0.0028 (4)	−0.0017 (5)
C6	0.0163 (6)	0.0211 (6)	0.0159 (6)	0.0010 (5)	−0.0009 (5)	−0.0056 (5)
C7	0.0155 (6)	0.0306 (7)	0.0217 (6)	−0.0043 (5)	0.0059 (5)	−0.0076 (5)
C8	0.0248 (7)	0.0261 (7)	0.0204 (6)	−0.0070 (6)	0.0097 (5)	−0.0034 (5)
C9	0.0246 (7)	0.0162 (6)	0.0157 (6)	−0.0026 (5)	0.0045 (5)	−0.0029 (5)
C10	0.0456 (9)	0.0204 (7)	0.0217 (7)	0.0052 (6)	−0.0018 (6)	0.0041 (5)
C11	0.0242 (7)	0.0172 (6)	0.0207 (6)	0.0055 (5)	−0.0039 (5)	0.0016 (5)
C12	0.0279 (8)	0.0373 (9)	0.0320 (8)	0.0138 (7)	−0.0028 (6)	−0.0102 (7)
C13	0.0122 (6)	0.0159 (6)	0.0163 (6)	0.0035 (4)	0.0007 (4)	−0.0002 (4)
C14	0.0138 (6)	0.0163 (6)	0.0174 (6)	0.0010 (5)	−0.0009 (5)	−0.0028 (5)
C15	0.0192 (6)	0.0242 (7)	0.0160 (6)	0.0026 (5)	0.0021 (5)	−0.0002 (5)
C16	0.0174 (6)	0.0223 (7)	0.0232 (6)	0.0006 (5)	0.0058 (5)	0.0027 (5)
C17	0.0138 (6)	0.0194 (6)	0.0270 (7)	−0.0009 (5)	0.0021 (5)	−0.0031 (5)
C18	0.0142 (6)	0.0194 (6)	0.0181 (6)	0.0019 (5)	−0.0005 (5)	−0.0031 (5)

### Geometric parameters (Å, °)

**Table:**

Cl1—C6	1.7415 (14)	C8—H8	0.9500
Cl2—C14	1.7405 (13)	C10—H10A	0.9800
O1—C11	1.2095 (17)	C10—H10B	0.9800
N1—C1	1.3181 (19)	C10—H10C	0.9800
N1—C9	1.3713 (17)	C11—C12	1.4996 (19)
C1—C2	1.4297 (18)	C12—H12A	0.9800
C1—C10	1.5055 (19)	C12—H12B	0.9800
C2—C3	1.3722 (17)	C12—H12C	0.9800
C2—C11	1.5091 (19)	C13—C14	1.3956 (17)
C3—C4	1.4250 (17)	C13—C18	1.3997 (18)
C3—C13	1.4932 (17)	C14—C15	1.3912 (18)
C4—C5	1.4137 (17)	C15—C16	1.3874 (19)
C4—C9	1.4186 (17)	C15—H15	0.9500
C5—C6	1.3649 (17)	C16—C17	1.3860 (19)
C5—H5	0.9500	C16—H16	0.9500
C6—C7	1.4077 (18)	C17—C18	1.3871 (18)
C7—C8	1.371 (2)	C17—H17	0.9500
C7—H7	0.9500	C18—H18	0.9500
C8—C9	1.4150 (19)		
			
C1—N1—C9	118.31 (11)	C1—C10—H10C	109.5
N1—C1—C2	122.63 (12)	H10A—C10—H10C	109.5
N1—C1—C10	117.20 (13)	H10B—C10—H10C	109.5
C2—C1—C10	120.16 (13)	O1—C11—C12	122.95 (13)
C3—C2—C1	120.06 (12)	O1—C11—C2	120.52 (12)
C3—C2—C11	120.27 (12)	C12—C11—C2	116.51 (12)
C1—C2—C11	119.66 (11)	C11—C12—H12A	109.5
C2—C3—C4	118.32 (11)	C11—C12—H12B	109.5
C2—C3—C13	120.71 (11)	H12A—C12—H12B	109.5
C4—C3—C13	120.96 (11)	C11—C12—H12C	109.5
C5—C4—C9	119.40 (12)	H12A—C12—H12C	109.5
C5—C4—C3	122.74 (11)	H12B—C12—H12C	109.5
C9—C4—C3	117.86 (11)	C14—C13—C18	117.70 (11)
C6—C5—C4	119.41 (12)	C14—C13—C3	122.28 (11)
C6—C5—H5	120.3	C18—C13—C3	120.00 (11)
C4—C5—H5	120.3	C15—C14—C13	121.65 (12)
C5—C6—C7	122.21 (12)	C15—C14—Cl2	118.18 (10)
C5—C6—Cl1	118.85 (10)	C13—C14—Cl2	120.13 (10)
C7—C6—Cl1	118.94 (10)	C16—C15—C14	119.32 (12)
C8—C7—C6	118.96 (12)	C16—C15—H15	120.3
C8—C7—H7	120.5	C14—C15—H15	120.3
C6—C7—H7	120.5	C17—C16—C15	120.22 (12)
C7—C8—C9	120.97 (12)	C17—C16—H16	119.9
C7—C8—H8	119.5	C15—C16—H16	119.9
C9—C8—H8	119.5	C16—C17—C18	119.95 (12)
N1—C9—C8	118.21 (12)	C16—C17—H17	120.0
N1—C9—C4	122.76 (12)	C18—C17—H17	120.0
C8—C9—C4	119.03 (12)	C17—C18—C13	121.15 (12)
C1—C10—H10A	109.5	C17—C18—H18	119.4
C1—C10—H10B	109.5	C13—C18—H18	119.4
H10A—C10—H10B	109.5		
			
C9—N1—C1—C2	−1.68 (19)	C7—C8—C9—C4	−1.06 (19)
C9—N1—C1—C10	179.92 (11)	C5—C4—C9—N1	−178.00 (11)
N1—C1—C2—C3	1.17 (19)	C3—C4—C9—N1	2.15 (18)
C10—C1—C2—C3	179.53 (12)	C5—C4—C9—C8	2.05 (18)
N1—C1—C2—C11	−177.73 (12)	C3—C4—C9—C8	−177.80 (11)
C10—C1—C2—C11	0.63 (18)	C3—C2—C11—O1	102.83 (15)
C1—C2—C3—C4	1.04 (17)	C1—C2—C11—O1	−78.27 (17)
C11—C2—C3—C4	179.94 (11)	C3—C2—C11—C12	−78.41 (16)
C1—C2—C3—C13	179.73 (11)	C1—C2—C11—C12	100.49 (15)
C11—C2—C3—C13	−1.37 (18)	C2—C3—C13—C14	110.11 (14)
C2—C3—C4—C5	177.58 (11)	C4—C3—C13—C14	−71.23 (16)
C13—C3—C4—C5	−1.11 (18)	C2—C3—C13—C18	−68.30 (16)
C2—C3—C4—C9	−2.57 (17)	C4—C3—C13—C18	110.35 (14)
C13—C3—C4—C9	178.74 (11)	C18—C13—C14—C15	0.86 (18)
C9—C4—C5—C6	−1.52 (18)	C3—C13—C14—C15	−177.59 (12)
C3—C4—C5—C6	178.33 (11)	C18—C13—C14—Cl2	178.64 (9)
C4—C5—C6—C7	−0.03 (19)	C3—C13—C14—Cl2	0.19 (17)
C4—C5—C6—Cl1	−179.77 (9)	C13—C14—C15—C16	−1.16 (19)
C5—C6—C7—C8	1.0 (2)	Cl2—C14—C15—C16	−178.98 (10)
Cl1—C6—C7—C8	−179.22 (10)	C14—C15—C16—C17	0.3 (2)
C6—C7—C8—C9	−0.5 (2)	C15—C16—C17—C18	0.8 (2)
C1—N1—C9—C8	179.94 (12)	C16—C17—C18—C13	−1.13 (19)
C1—N1—C9—C4	−0.02 (19)	C14—C13—C18—C17	0.29 (18)
C7—C8—C9—N1	178.98 (12)	C3—C13—C18—C17	178.78 (11)

### Hydrogen-bond geometry (Å, °)

**Table:**

*D*—H···*A*	*D*—H	H···*A*	*D*···*A*	*D*—H···*A*
C18—H18···O1^i^	0.95	2.59	3.2460 (17)	127

Symmetry codes: (i) −*x*+1, −*y*+2, −*z*+2.
